# The pulsating soft coral *Xenia umbellata* shows high resistance to warming when nitrate concentrations are low

**DOI:** 10.1038/s41598-022-21110-w

**Published:** 2022-10-06

**Authors:** Bianca Thobor, Arjen Tilstra, David G. Bourne, Karin Springer, Selma Deborah Mezger, Ulrich Struck, Franziska Bockelmann, Lisa Zimmermann, Ana Belén Yánez Suárez, Annabell Klinke, Christian Wild

**Affiliations:** 1 Faculty of Biology and Chemistry, Department of Marine Ecology, University of Bremen, UFT Building, Leobener Str. 6, 28359 Bremen, Germany; 2 College of Science and Engineering, James Cook University, 1 Angus Smith Drive, Douglas, QLD 4814 Australia; 3 Australian Institute of Marine Science, Cape Ferguson, Townsville, QLD 4810 Australia; 4 Faculty of Biology and Chemistry, Marine Botany, University of Bremen, NW2 Building, Leobener Str. 5, 28359 Bremen, Germany; 5 Museum Für Naturkunde, Leibniz Institute for Evolution and Biodiversity Science, Invalidenstr. 43, 10115 Berlin, Germany; 6 Department of Earth Sciences, Free University Berlin, Malteserstr. 74-100, Haus D, 12249 Berlin, Germany

**Keywords:** Ecophysiology, Macroecology, Stable isotope analysis, Tropical ecology

## Abstract

The resistance of hard corals to warming can be negatively affected by nitrate eutrophication, but related knowledge for soft corals is scarce. We thus investigated the ecophysiological response of the pulsating soft coral *Xenia umbellata* to different levels of nitrate eutrophication (control = 0.6, medium = 6, high = 37 μM nitrate) in a laboratory experiment, with additional warming (27.7 to 32.8 °C) from days 17 to 37. High nitrate eutrophication enhanced cellular chlorophyll *a* content of Symbiodiniaceae by 168%, while it reduced gross photosynthesis by 56%. After additional warming, polyp pulsation rate was reduced by 100% in both nitrate eutrophication treatments, and additional polyp loss of 7% d^−1^ and total fragment mortality of 26% was observed in the high nitrate eutrophication treatment. Warming alone did not affect any of the investigated response parameters. These results suggest that *X. umbellata* exhibits resistance to warming, which may facilitate ecological dominance over some hard corals as ocean temperatures warm, though a clear negative physiological response occurs when combined with nitrate eutrophication. This study thus confirms the importance of investigating combinations of global and local factors to understand and manage changing coral reefs.

## Introduction

Anthropogenically caused accumulation of carbon dioxide (CO_2_) results in excess heat in the atmosphere, which is absorbed by the ocean and ultimately causes ocean warming^1^. Due to this, increased frequency of coral bleaching events is predicted over the next century, leaving less time for recovery^2^. The loss and damage to coral reef ecosystems has serious economic consequences on the livelihoods of those who depend on fisheries and tourism^2^.

Coral bleaching is a stress response that is commonly described by a loss of algal symbiont pigmentation, loss of algal symbiont cell number or a combination of both, which in turn changes coral colour and constitutes the breakdown of the symbiosis^3,4^. This stress response can be triggered by seawater warming^5^, which has been connected to increased production of reactive oxygen species (ROS) by algal symbionts^3^ and shifts in nutrient cycling between coral host and algal symbiont^6^. Fitness of shallow water corals depends on a stable nutritional exchange with their algal symbionts^6^ of the family Symbiodiniaceae^7^. When this symbiosis is disrupted, a reduction in the organisms energy budget can affect coral host health and subsequently cause an increase in mortality^8^.

The cell numbers of the endosymbiotic algae populations within coral tissues are controlled through nitrogen (N) limitation by the coral host^8^ and concentrated discharge of coastal wastewater may result in excess of dissolved inorganic nutrients such as nitrate^9^, one of the main forms of inorganic N in wastewater impacted sites^10^. It is now clear that such anthropogenic eutrophication causes disproportionate nutrient availability which can affect the stability of the coral-algae symbiosis (as reviewed by Morris et al*.*^11^). Excess N may increase proliferation of algal symbiont cells^12^, but may also increase the cellular demand for other nutrients which can potentially lead to relative phosphorus (P) starvation^13^. Nitrate assimilation in particular is associated with higher energetic costs than ammonium in plants^14^ and caused reduced photosynthesis in a hard coral, while ammonium enhanced photosynthesis^15^.

Predicted scenarios of ocean warming and increased inorganic eutrophication will affect most nearshore coral reefs worldwide simultaneously^16^. Furthermore, documented decline in coral cover varies between regions, indicating that local factors such as water quality may play a role in determining responses to ocean warming for some coral taxa^17,18^. Due to these increasing synergistic pressures facing coral reefs, studies that investigate the interaction of eutrophication and ocean warming are becoming a priority^9,19^. Previous studies show that the thermally-induced bleaching response may be exacerbated when combined with local eutrophication^20,21^. Synergistic effects of warming and eutrophication on corals can result for example from P starvation^9,15^, increased parasitic activity of algal symbionts^22^, or increased oxidative stress^23^. A recent review by Morris et al*.*^11^ summarises the effects of nutrient stress on corals and its implications for thermal tolerance. Most studies investigating the effects of temperature and eutrophication on corals have focused on scleractinian corals^20^ with fewer studies investigating these combined effects on soft corals^24^. Community shifts from hard coral to soft coral dominance have been observed under a variety of disturbance regimes^25,26^. Thus, soft corals may become more abundant on some reefs in the future which has implications for whole ecosystems, since soft corals do not have the ecosystem engineer characteristics of hard corals in supporting reef fish assemblages through structural complexity^27,28^. However, Epstein & Kingsford^29^ found increasing fish diversity with increasing soft coral, but not hard coral cover, for a reef in the Great Barrier Reef (GBR) and highlighted that soft corals could have higher ecological importance than previously assumed. Knowledge about processes benefiting soft corals under certain environmental conditions is needed to better understand and predict future coral reef community compositions.

**Figure 1:**
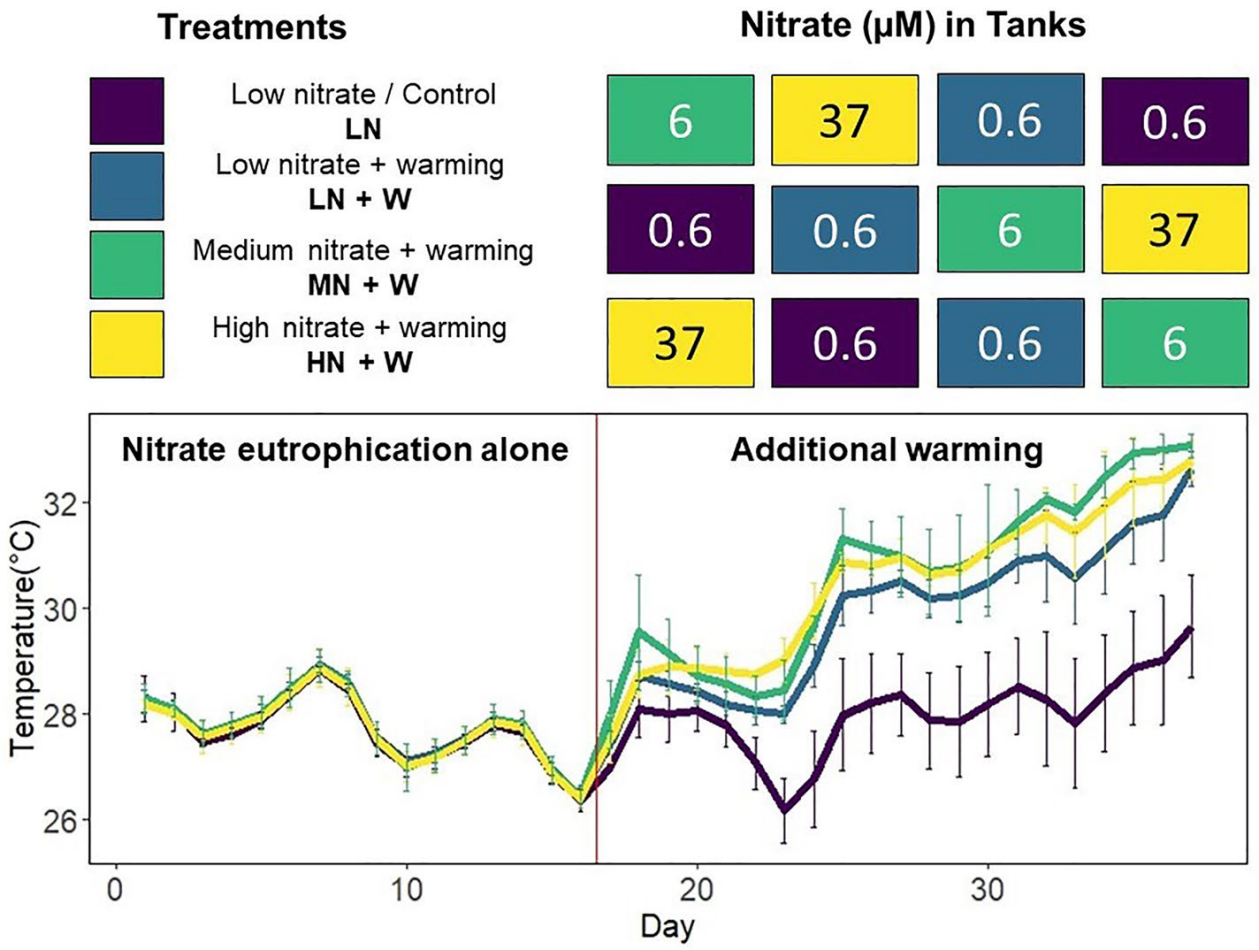
Experimental design with development of temperatures per treatment. Tanks were arranged in the depicted order vertically, with four tanks on every level. The experiment lasted 37 days, and temperatures were increased gradually from day 17 in all but the low nitrate (LN) control tanks. During the first 16 days of the experiment, both low nitrate treatments (LN and LN + W) were exposed to the same conditions. This changed as temperatures in the LN + W treatment increased together with the MN + W and HN + W treatments.

To improve our understanding of the effects of inorganic eutrophication and warming on soft corals, this study aimed to answer the following research questions: (i) How does nitrate eutrophication affect *X. umbellata*? (ii) How does chronic nitrate eutrophication affect the response of *X. umbellata* to warming? We also discuss whether *X. umbellata* is more or less resistant to nitrate eutrophication and warming than hard corals, and the implications for coastal management. *Xenia umbellata* was used because this pulsating soft coral is common and widespread in the Indo-Pacific^30,31^ and the Red Sea^32^. Because a fully factorial experimental design was not possible with our aquarium facilities, we chose to primarily investigate the effects of nitrate eutrophication on the resistance of *X. umbellata* to warming. For this, *X. umbellata* was exposed to medium (6 μM) and high (37 μM) nitrate eutrophication (controls ~ 0.6 μM). After 17 days, temperatures were gradually increased from an average of 27.7 ± 0.7 °C from days 1–16, to 32.8 ± 0.3 °C on day 37 in all but control tanks (a total increase of 5 °C over 22 days; see Fig. 1 for detailed experimental design). To assess the coral health status in response to nitrate eutrophication and /or warming, we measured coral colony survival, growth rate, polyp pulsation rate, gross photosynthesis (P_gross_), respiration (R), algal symbiont cell density, chlorophyll *a* (chl *a*) content, coral colouration, and elemental and stable isotope composition (to provide information about nutrient uptake and utilization).

## Results

### Colony survival and growth rate

**Figure 2:**
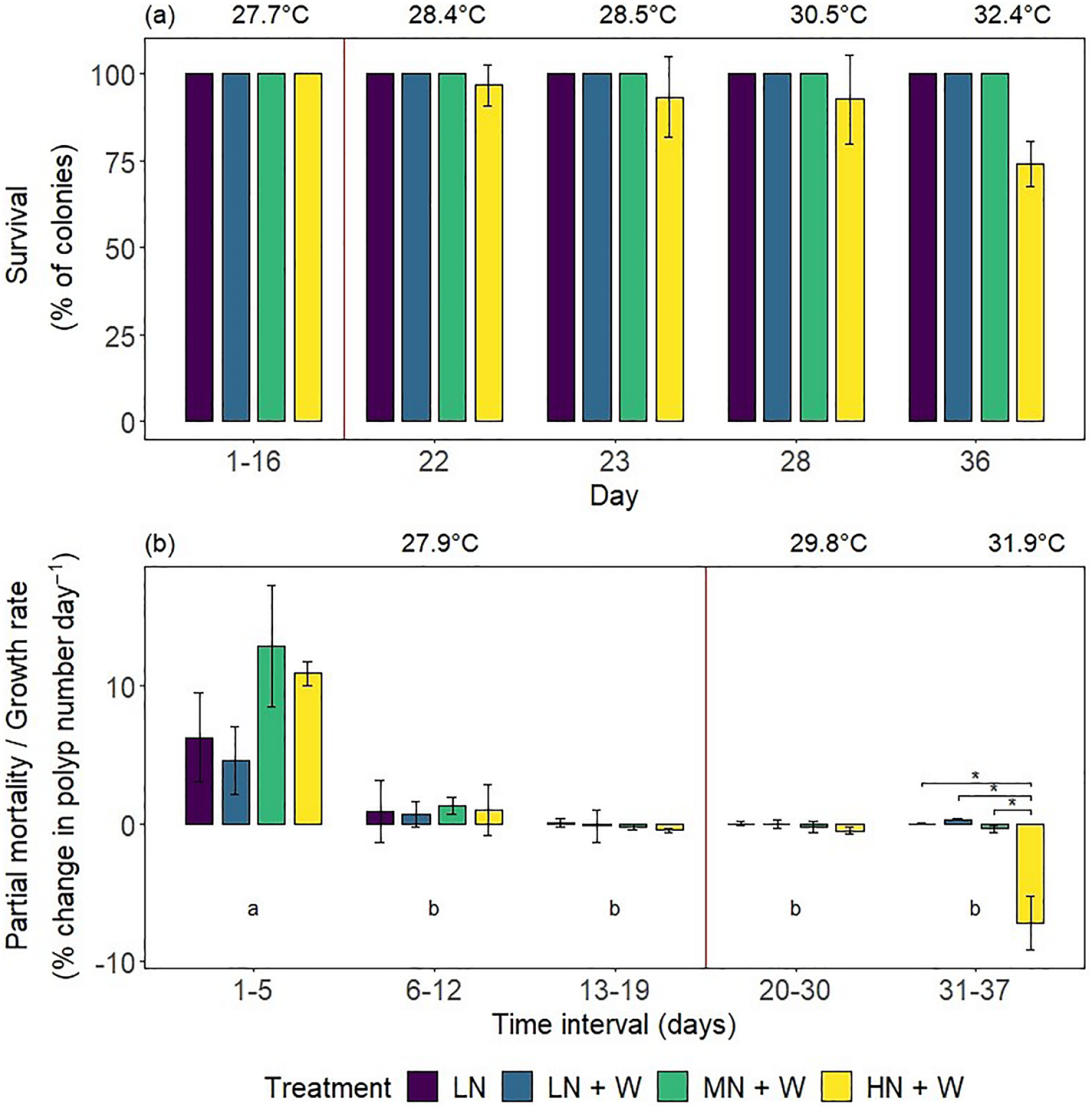
(**a**) Percent survival and (**b**) growth rates of *Xenia umbellata* colonies from control tanks with low nitrate (LN, ~ 0.6 μM) and three treatments: LN + W = low nitrate (~ 0.6 μM) + warming from day 17; MN + W = medium nitrate eutrophication (~ 6 μM) + warming from day 17; HN + W = high nitrate eutrophication (~ 37 μM) + warming from day 17. Error bars represent standard deviations of three replicates. Temperatures represent mean temperatures of the respective days or intervals, excluding controls. Different letters in (**b**) indicate significant differences between days (pwc, Bonferroni adjustment, *t*-test, *p* < 0.05). Asterisks indicate significant differences between treatments within days (pwc, Bonferroni adjustment, *t*-test, * = *p* < 0.05). For (**a**), only days with recorded colony mortalities were plotted (except day 1–16). No *post-hoc* analysis could be performed for (**a**) due to lack of variance within groups where all replicate tanks displayed 100% survival.

The treatment effect on colony survival was significant (Wald-type statistic = 4.14, *p* < 0.05; ANOVA-type statistic = 4.14, *p* < 0.05). Survival was only affected by high nitrate eutrophication (HN + W) with additional warming (Fig. 2a). The first mortality was observed on day 22 (at 28.4 °C). On day 36 (at 32.4 °C), the average survival was 74%.

The overall treatment effect on growth rates was not significant. However, colonies exposed to high nitrate eutrophication and additional warming (HN + W) displayed partial mortality (mortality of some colony polyps, measured as negative growth rate), averaging 7.2 ± 4.1% polyp loss d^-1^ during the last week of the experiment, with a mean temperature of 31.9 °C (Fig. 2b). This partial mortality was significantly higher than observed for all other treatments and controls (pwc, Bonferroni adjustment, *t*-test, *p* < 0.05). Colony growth rates decreased in all tanks shortly after the start of the experiment, and the time interval of the experiment significantly affected growth rates (2-way mixed ANOVA, *F* = 29.21, *p* < 0.001).

### Polyp pulsation rate

**Figure 3:**
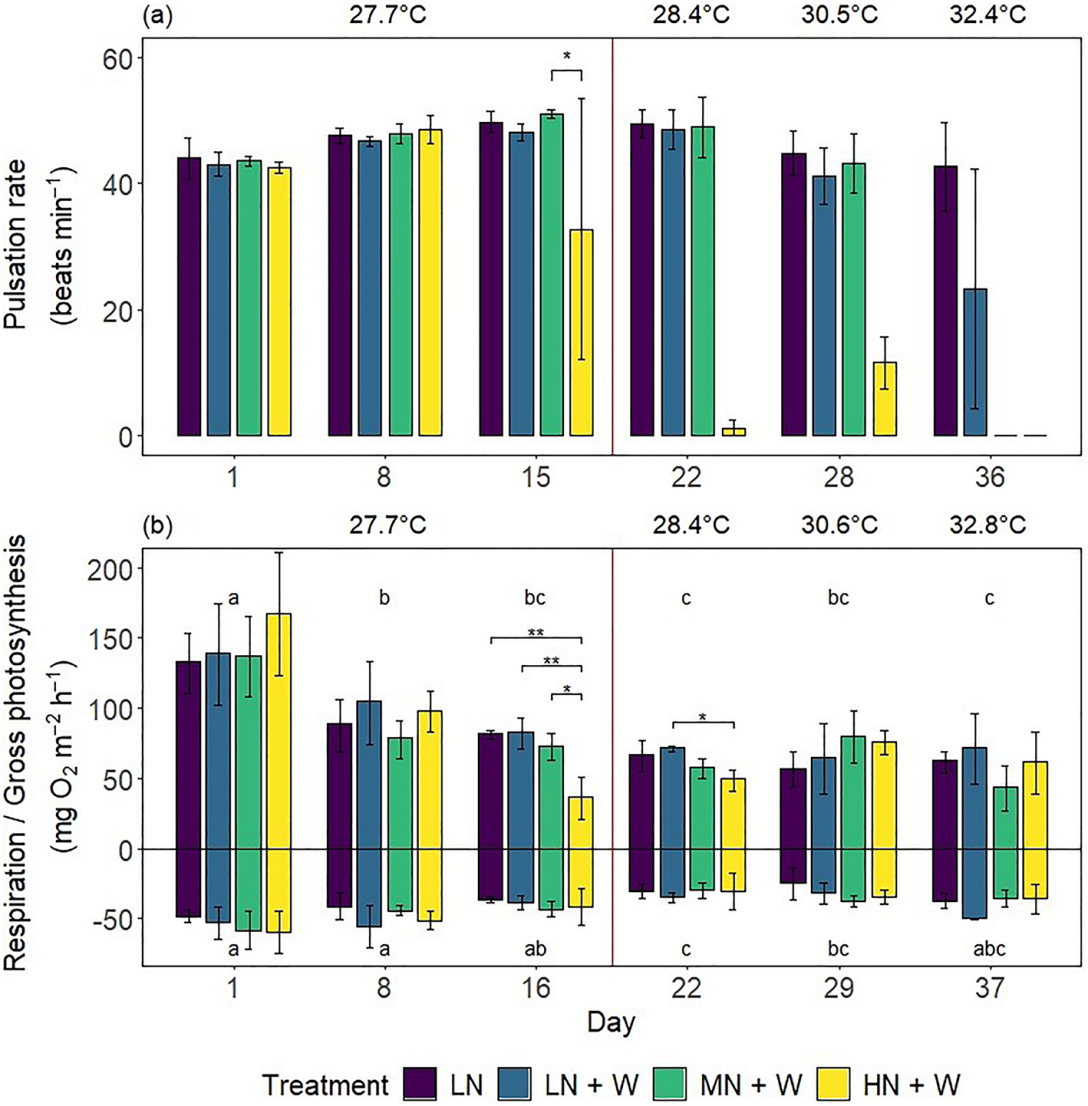
(**a**) Pulsation rates and (**b**) gross photosynthesis (P_gross_) and respiration (R) of *Xenia umbellata* colonies from control tanks with low nitrate (LN, ~ 0.6 μM) and three treatments: LN + W = low nitrate (~ 0.6 μM) + warming from day 17; MN + W = medium nitrate eutrophication (~ 6 μM) + warming from day 17; HN + W = high nitrate eutrophication (~ 37 μM) + warming from day 17. Error bars represent standard deviations of three replicates. Temperatures represent mean temperatures of the respective days, excluding controls. Different letters in (**a**) indicate significant differences between days (pwc, Bonferroni adjustment, *t*-test, *p* < 0.05). Asterisks represent significant differences between treatments within days (pwc, Bonferroni adjustment, (**a**) *t*-test & (**b**) Dunn's test, ** = *p* < 0.005, * = *p* < 0.05).

Overall, the effect of treatment on pulsation rates varied significantly between days of the experiment (Wald-type statistic = 81.87, p < 0.001; ANOVA-type statistic = 3.52, *p* < 0.01). Pulsation rates of colonies exposed to high nitrate eutrophication (HN + W) were reduced by 36% compared to the medium nitrate eutrophication treatment (MN + W) after 15 days (Fig. 3a and Table 1; pwc, Bonferroni adjustment, Dunn's test, *p* < 0.05), but they did not significantly differ from controls. On day 22, after additional warming, pulsation rates of colonies exposed to high nitrate eutrophication decreased by 97% compared to controls (at 28.4 °C). With medium nitrate eutrophication (MN + W), pulsation rates remained stable until day 28 (at 30.5 °C) and dropped to zero during the last week of the experiment (at > 30.6 °C). At the end of the experiment (day 36 at 32.4 °C), pulsation could not be observed under medium or high nitrate eutrophication. Corals exposed to warming alone (LN + W) exhibited no significant reduction in pulsation rates.

**Table 1:** Effect size (%) of ecophysiological parameters relative to controls (LN; ~ 0.6 μM NO_3_, no warming). Bold values indicate significant differences to controls (LN) and values in brackets indicate effect size relative to low nitrate-treated colonies exposed to warming (LN + W). P_gross_ = gross photosynthesis, R = respiration, C:N = carbon to nitrogen ratio, %N/C = percent nitrogen/carbon of tissue dry weight.

Warming	NO_3_ (μM)	Pulsation rate	P_gross_	R	Symbiont density	Chl *a* content	Colour score			C:N	% N	% C
							Red	Green	Blue			
- (Day 15–17 )	6	+ 3	−11	+ 16	−17	+ 75	0	0	0	−10	+ 6	−12
	37	−34	**−56**	+ 19	−10	** + 168**	−4	+ 16	+ 29	−28	+ 23	**−18**
+ 5 °C (Day 37)	0.6	−45	+ 15	+ 35	−12	+ 29	0	0	0	−9	+ 18	+ 8
	6	−100 (−100)	−30 (−40)	−4 (−29)	−11 (+ 1)	+ 59 (+ 23)	−1 (−1)	+ 2 (+ 2)	+ 4 (+ 4)	−16 (−8)	+ 22 (+ 3)	+ 2 (−6)
	37	−100 (−100)	0 (−14)	−3 (−28)	+ 36 (+ 54)	+ 81 (+ 40)	−6 (−6)	+ 25 (+ 25)	+ 44 (+ 44)	**−30** (−24)	** + 58** **(+ 34)**	+ 10 (+ 2)

### Coral holobiont gross photosynthesis and respiration rates

The overall effect of treatment on P_gross_ was not significant. Colonies exposed to high nitrate eutrophication (HN + W) exhibited reduced P_gross_ (by 56%) compared to controls after 16 days (Fig. 3b and Table 1; pwc, Bonferroni adjustment, *t*-test, *p* < 0.01). With additional warming, no significant treatment effect was observed. P_gross_ values for all treatments varied significantly over time (2-way mixed ANOVA, *F* = 29.35, *p* < 0.001) with highest values on day one and lowest values on day 22 and 37.

Treatments did not significantly affect R, though there was a trend of declining R in all treatments throughout the experiment (2-way mixed ANOVA, *F* = 12.08, *p* < 0.001) with highest R on day one and eight and lowest R on day 22 (Fig. 3b). Spearman’s correlation analysis revealed a significant negative correlation of P_gross_ and R (Supplementary Figure S1, *r*_*S*_ = −0.63, n = 72, *p* < 0.001).

### Algal symbiont cell density, chlorophyll a content, and coral colouration

**Figure 4:**
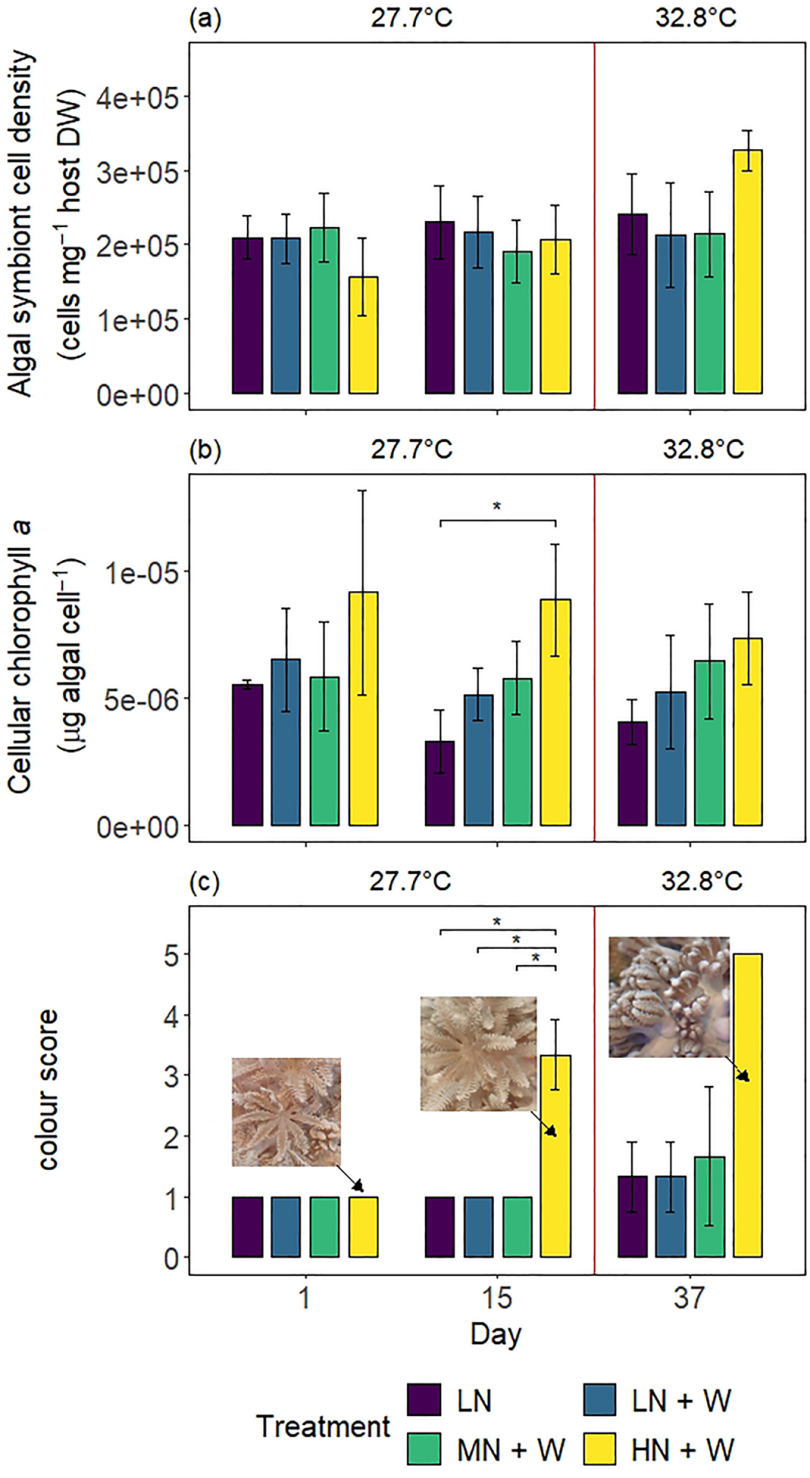
(**a**) Algal symbiont cell density, (**b**) chlorophyll *a* content standardized to cell density, and (**c**) colour scores (definitions in Supplementary Table S2, see also Supplementary Fig. S3) of *Xenia umbellata* colonies from control tanks with low nitrate (LN, ~ 0.6 μM) and three treatments: LN + W = low nitrate (~ 0.6 μM) + warming from day 17; MN + W = medium nitrate eutrophication (~ 6 μM) + warming from day 17; HN + W = high nitrate eutrophication (~ 37 μM) + warming from day 17. Error bars represent standard deviations of three replicates, with one exception of two replicates for the LN + W treatment on day one for both, (**a**) and (**b**). Temperatures represent mean temperatures of the respective days, excluding controls (LN). Asterisks represent significant differences between treatments within days (pwc, Bonferroni adjustment, *t*-test (**b**) or Dunn’s test (**c**), * = *p* < 0.05). Post-hoc test in (**c**) was conducted excluding day 1. Pictures in (**c**) show representative polyps of one identical *X. umbellata* colony in the high nitrate treatment at the respective time points (as indicated by arrows). Images in (**c**) by Lisa Zimmermann.

None of the treatments resulted in significant differences in algal symbiont cell densities throughout the experiment (Fig. 4a and Table 1).

Treatments significantly affected algal symbiont chl *a* content (2-way ANOVA, *F* = 6.648, *p* < 0.01). Colonies exposed to high nitrate eutrophication (HN + W) exhibited 168% higher chl *a* concentrations than controls (LN) after 15 days (Fig. 4b and Table 1; pwc, Bonferroni adjustment, *t*-test, *p* < 0.05). Additional warming did not result in significant differences between treatments by the end of the experiment.

The effect of treatment on colour scores varied significantly between days of the experiment (Wald-type statistic = 731.95, *p* < 0.001; ANOVA-type statistic = 3.59, *p* < 0.05). The colour score of corals exposed to high nitrate eutrophication (HN + W) increased from 1.0 to 3.3 after 15 days (Fig. 4c). This was significantly higher compared to all other treatments and controls (pwc, Bonderroni adjustment, Dunn’s test, *p* < 0.05). Based on the definition of each colour score (Supplementary Table S2), this was equivalent to a 16% and 29% increase in green and blue values, respectively, and reduced red values by 4% (Table 1). After additional warming, the colour score of all corals exposed to high nitrate eutrophication was five, which was not significantly different from other treatments or controls. The change in colour score from one to five was equivalent to increased green and blue values by 25% and 44%, respectively, and reduced red values by 6%.

### Nitrogen and carbon elemental composition, and stable isotope ratios

**Figure 5:**
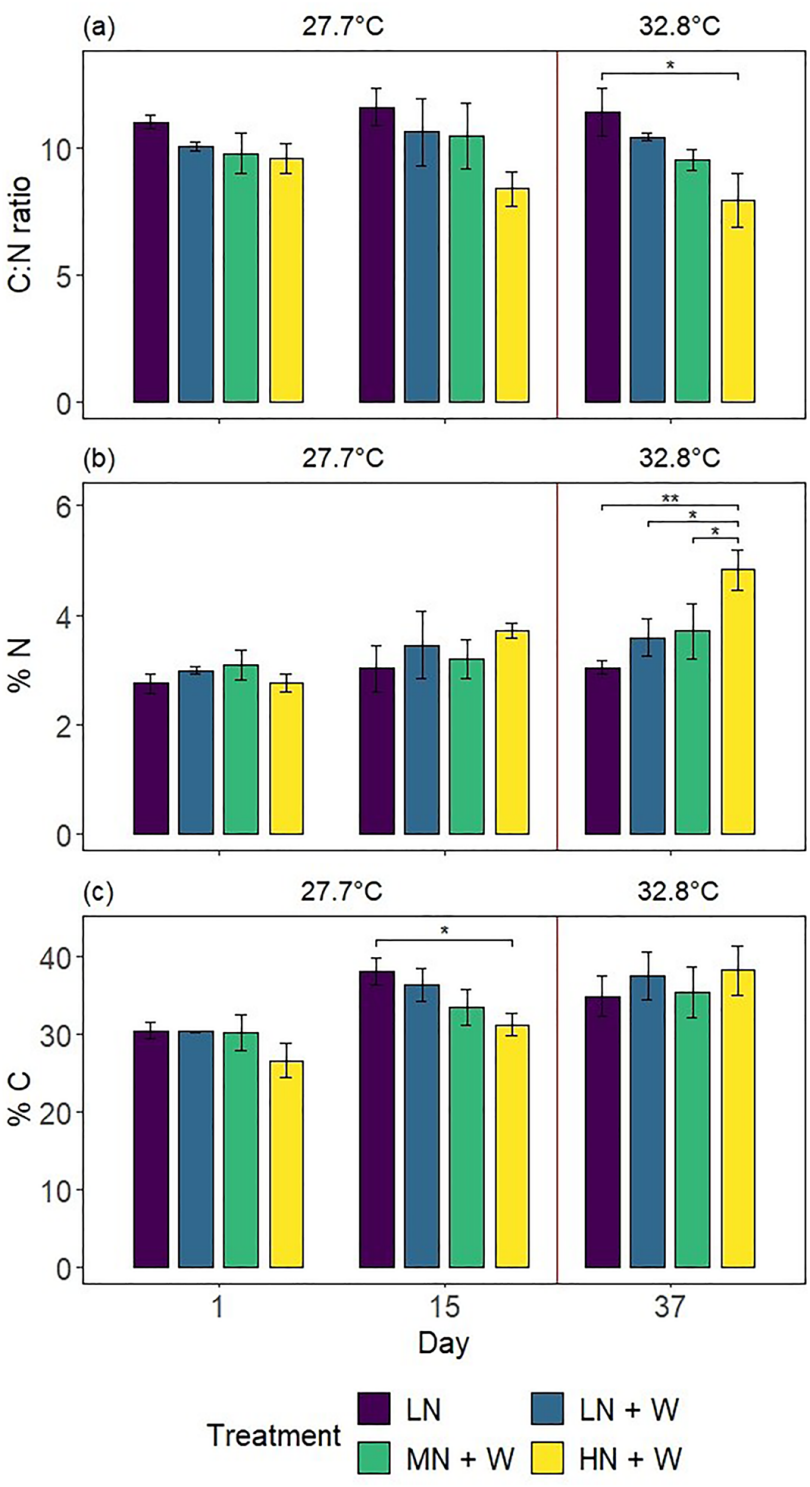
(**a**) Carbon to nitrogen ratio, (**b**) percent nitrogen and (**c**) percent carbon per dry weight of *Xenia umbellata* colonies from control tanks with low nitrate (LN, ~ 0.6 μM) and three treatments: LN + W = low nitrate (~ 0.6 μM) + warming from day 17; MN + W = medium nitrate eutrophication (~ 6 μM) + warming from day 17; HN + W = high nitrate eutrophication (~ 37 μM) + warming from day 17. Error bars represent standard deviations of three replicates, with exceptions of two replicates for controls (LN) on day 15 (**a** & **c**) and the LN + W treatment on day 37 (**a**, **b** & **c**). Temperatures represent mean temperatures of the respective days, excluding controls (LN). Asterisks indicate significant differences between treatments within days (pwc, Bonferroni adjustment, *t*-test, ** = *p* < 0.005, * = *p* < 0.05).

Treatments significantly affected the ratios of total carbon (C) to total N (C:N ratios) of coral colonies (2-way ANOVA, *F* = 15.756, *p* < 0.001). Nitrate eutrophication for 15 days alone did not affect C:N ratios (Fig. 5a and Table 1). After additional warming, colonies exposed to high nitrate eutrophication (HN + W) displayed 30% lower C:N ratios compared to controls (pwc, Bonferroni adjustment, *t*-test, *p* < 0.01), and 24% lower C:N ratios compared to colonies exposed to warming alone (LN + W; *p* > 0.05).

The effect of treatment on percent N content of coral colonies varied between days of the experiment (2-way ANOVA, *F* = 4.294, *p* < 0.01). Percent N contents were not affected after 15 days of nitrate eutrophication alone (Fig. 5b and Table 1). After additional warming, corals exposed to high nitrate eutrophication (HN + W) revealed 58% higher N contents compared to controls (LN; pwc, Bonferroni adjustment, *t*-test, *p* < 0.005), 34% higher N contents compared to colonies exposed to warming alone (LN + W; *p* < 0.05), and 30% higher N contents compared to colonies exposed to medium nitrate eutrophication and warming (MN + W; *p* < 0.05).

The effect of treatment on percent C contents of coral colonies varied between days of the experiment (2-way ANOVA, *F* = 2.821, *p* < 0.05). Coral colonies exposed to high nitrate eutrophication (HN + W) displayed 18% lower C contents compared to controls (LN) after 15 days (Fig. 5c and Table 1; pwc, Bonferroni adjustment, *t*-test, *p* < 0.05), with no significant effects after additional warming.

**Figure 6:**
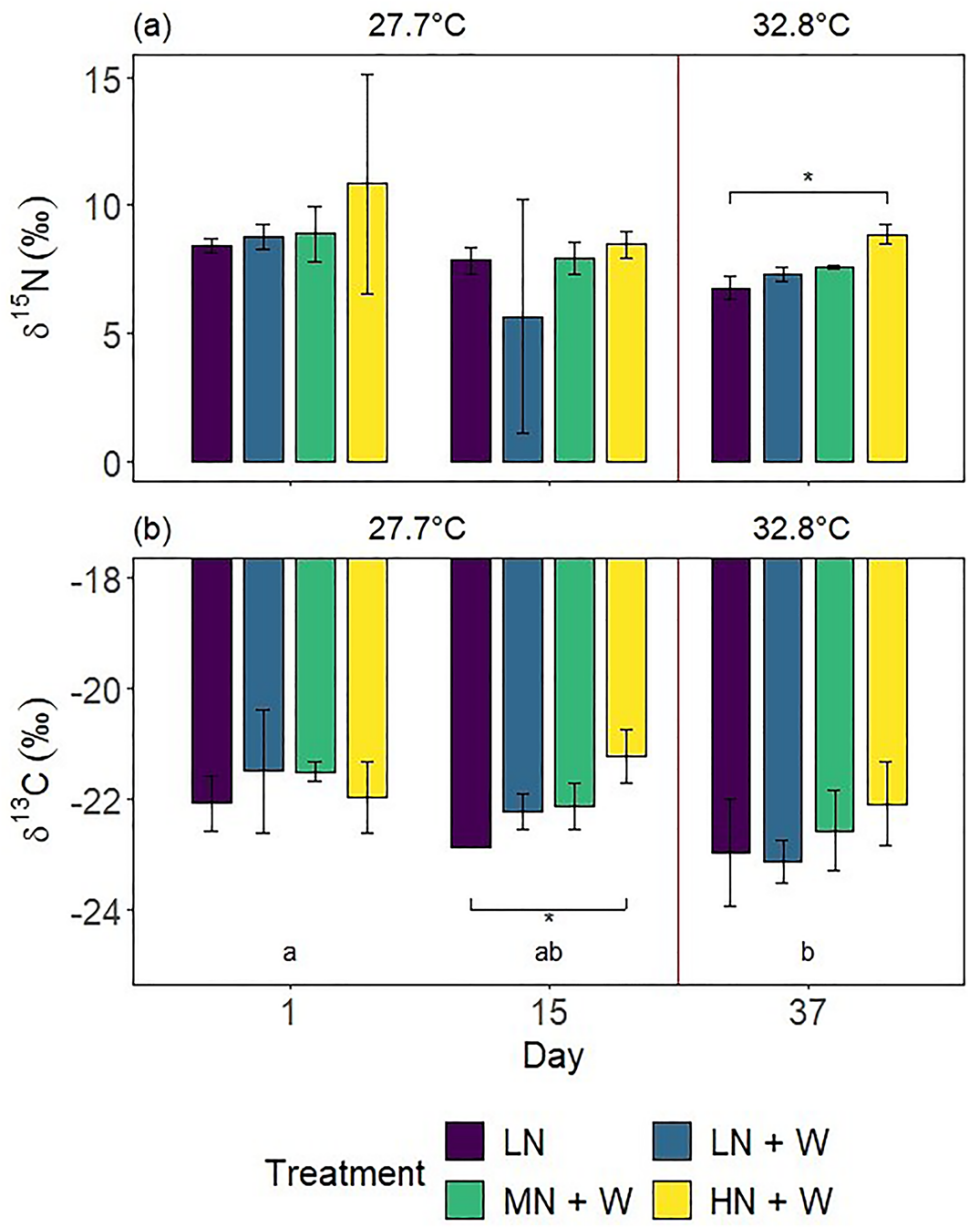
(**a**) Nitrogen and (**b**) carbon stable isotope ratios of *Xenia umbellata* colonies from control tanks with low nitrate (LN, ~ 0.6 μM) and three treatments: LN + W = low nitrate (~ 0.6 μM) + warming from day 17; MN + W = medium nitrate eutrophication (~ 6 μM) + warming from day 17; HN + W = high nitrate eutrophication (~ 37 μM) + warming from day 17. Error bars represent standard deviations of three replicates, with exceptions of two replicates for controls (LN) on day 15 (**b**) and the LN + W treatment on day 37 (**a** & **b**). Temperatures represent mean temperatures of the respective days, excluding controls. Different letters in (**b**) indicate significant differences between days (pwc, Bonferroni adjustment, *t*-test, *p* < 0.05). Asterisks indicate significant differences between treatments within days (pwc, Bonferroni adjustment, (**a**) Dunn’s test, (**b**) *t*-test, * = *p* < 0.05).

Treatment and day of the experiment both significantly affected δ^15^N values of coral colonies (2-way ANOVA, Treatment: *F* = 3.28, *p* < 0.05; Day: *F* = 5.04, *p* < 0.05). The δ^15^N values were not affected after 15 days of nitrate eutrophication alone (Fig. 6a), averaging 8.4 ± 2.3 ‰. After additional warming, colonies exposed to high nitrate eutrophication (HN + W) exhibited 31% higher δ^15^N values than controls (LN; pwc, Bonferroni adjustment, Dunn’s test, *p* < 0.05) and 21% higher δ^15^N values compared to colonies exposed to warming alone (LN + W; *p* > 0.05).

The overall treatment effect on δ^13^C values was not significant. Colonies exposed to high nitrate eutrophication (HN + W) displayed 7% higher δ^13^C values compared to controls (LN) after 15 days (Fig. 6b and Table 1; pwc, Bonferroni adjustment, *t*-test, *p* < 0.05). No significant treatment effect was observed after additional warming. The δ^13^C values decreased over time, with day of the experiment significantly affecting δ^13^C values (2-way ANOVA, *F* = 5.557, *p* < 0.05).

## Discussion

### Nitrate eutrophication alone did not significantly affect pulsation rate, growth rate or survival

Pulsation rates were reduced by 34% with high nitrate eutrophication after 15 days, though not significantly compared to controls. In pulsating soft corals, reduced pulsation rates can also lead to reduced photosynthesis, as pulsation normally enhances gas exchange, e.g. oxygen transport away from the coral's surface^33^. Thus, reduced pulsation rates may have caused reduced P_gross_ with high nitrate eutrophication, or vice versa. Furthermore, reduced P_gross_ can lead to reduced transfer of photosynthates to the coral host^15^. Resulting energy depletion of the coral host could have caused a reduction of the energy demanding pulsation to conserve energy for more vital processes. The additionally observed increase in δ^13^C values in colonies exposed to high nitrate eutrophication compared to controls on day 15 also indicates a shift in C metabolism. Increasing δ^13^C values may arise from increasing photosynthesis^34^, which was likely not the case in the present study, as P_gross_ declined while δ^13^C increased. Zooplankton is generally depleted of the heavier ^13^C isotope^34^ and thus, Grottoli et al*.*^35^ interpreted an increase in the δ^13^C value of two bleached hard corals to be caused by reduced heterotrophy. This may also explain the results of the present study, as reduced pulsation rates may affect the coral’s ability to filter feed^33^. While coral host growth rate in the present study was not affected by nitrate eutrophication and no mortality was observed after 15 days at ambient temperatures, growth rates decreased significantly across all treatments and controls, from an initial five polyps d^-1^ to almost zero. In contrast, eutrophication often leads to declining coral growth rates in hard corals, especially with simultaneously declining photosynthesis^12^. The decline of growth rates observed for all treatments could be explained by reduced food availability of *X. umbellata* colonies during the experiment compared to prior aquarium conditions, where they were kept with other invertebrates and reef fish, potentially resulting in higher input of organic matter into the water relative to experimental conditions.

### High nitrate eutrophication enhanced coral pigmentation, while it reduced photosynthesis

Cellular chl *a* content was significantly enhanced under high nitrate eutrophication compared to controls after 15 days, while P_gross_ declined, and algal symbiont cell densities remained stable. These observations are contradictive to previous studies on hard corals, which found simultaneous increases of chl *a* and P_gross_ with N eutrophication^36,37^, generally explained by a release from N limitation of algal symbionts^8,15^. However, Ezzat et al*.*^15^ also found reduced P_gross_ with simultaneous increases in total chl *a* and algal symbiont cell densities in *Stylophora pistillata*, and explained this with the energy consuming process of nitrate reduction in the chloroplasts^38^. An additional explanation could be a limitation of photosynthesis by dissolved inorganic carbon (DIC) with increasing cellular chl *a* contents^39^. Release from N limitation can result in reduced energy supply to the coral host, as photosynthates are increasingly retained by the algal symbionts for their own growth^6,8,15^. This could affect the energy demanding CO_2_-concentration mechanisms (CCMs) by the coral host^39^. DIC limitation under continued irradiance can cause the production of ROS, and reduced photosynthetic rates even before algal symbiont expulsion^39^. Damage to chl *a* due to ROS could not be assessed, due to interference of chl *a* degradation products with the used method^40^. Furthermore, the colour of *X. umbellata* colonies exposed to high nitrate eutrophication changed distinctly, with increasing green and blue values (Supplementary Table S2). Change of colour in corals is often associated with coral bleaching. However, bleaching describes paling of the tissue as a result of loss of algal pigmentation or loss of algal symbionts from the coral host^3,4^, both of which were not observed in the present study. In contrast, tissue darkened while cellular chl *a* content increased.

The C:N ratios of *X. umbellata* tissue and algal symbionts remained above the canonical Redfield ratio of 6.625^41^ throughout the experiment (i.e., 6.72 and higher). In addition, phosphate starvation (e.g., caused by a high influx of N) may cause reduced photosynthesis under high environmental N:P ratios, especially with increasing algal symbiont populations^9,13^. High concentrations of nitrate (37 μM), and subsequent N:P ratios (176:1, based on phosphate concentrations of ≤ 0.21 μM, Supplementary Table S1) exceeding the Redfield ratio of 16:1, were accompanied with significantly reduced P_gross_ after 16 days while no effect on R was observed. However, in the present study, no effect was found on algal symbiont cell densities (Fig. 4a). Rather, algal symbiont cell densities remained stable and within the range expected for soft corals^42^, while cellular chl *a* content increased. This suggests a disturbance of photosynthesis rather than loss of algal symbionts to be the cause for reduced P_gross_. Thus, together with the high C:N ratios found in the current study, N may have remained the limiting nutrient throughout this study or it is possible that *X. umbellata* has mechanisms in place to effectively deal with high environmental N availability and/or algal symbiont P starvation. Pupier et al*.*^43^ found up to tenfold lower dissolved nitrogen assimilation rates in soft corals compared to hard corals, highlighting their differences in nutritional strategies which may benefit soft corals in eutrophic environments. Further investigation is required to specify the underlying cause of the reduced photosynthesis under nitrate eutrophication observed in the present study, with studies simulating phosphate eutrophication, also in combination with an N-source, to potentially indicate if algal symbionts of *X. umbellata* are prone to phosphate starvation.

Bednarz et al*.*^44^ found no effect on chl *a* content, P_gross_ or R in *Xenia spp.* after four weeks of ammonium eutrophication (20 μM), indicating potential different effects of ammonium and nitrate on xeniid corals. Nitrate reduction can act as an additional sink for reduction equivalents involved in photosynthesis, which was shown to reduce photosynthesis in *S. pistillata*, with ammonium having an opposite effect^15^. Furthermore, nitrate eutrophication in combination with warming caused increased oxidative stress and coral bleaching in *S. pistillata*, while ammonium eutrophication benefited the coral during warming^23^. Similarly, the resistance of *Turbinaria reniformis* to warming was increased with ammonium eutrophication^45^, but negatively affected by nitrate eutrophication without simultaneous P enrichment^46^. Further studies comparing the effects of nitrate and ammonium eutrophication on the response of *X. umbellata* to warming may reveal if nitrate eutrophication has particularly negative effects on the response of *X. umbellata* to warming, or if N eutrophication (and possibly P starvation) is responsible for the observed effects on thermal tolerance of the soft coral.

### Nitrate eutrophication negatively affected the resistance of *X. umbellata* to warming

Pulsation stopped with combined nitrate eutrophication (both medium and high concentrations) and warming at the end of the experiment, with additional increased partial mortality and 26% colony mortality with high nitrate eutrophication. In contrast, corals exposed to warming alone only exhibited a 45% reduction in pulsation rates relative to controls (not significant), with stable growth rates and no mortality. This strongly indicates negative effects of nitrate eutrophication, even at medium concentrations, on the resistance of *X. umbellata* to warming, while photophysiological parameters (algal symbiont cell density, pigmentation, photosynthesis) were not negatively affected. N eutrophication can increase the susceptibility of hard corals to warming, due to P starvation^9,15^, oxidative stress^23^, or increased parasitism of algal symbionts^22^. All of these explanations presume a decrease in photophysiological parameters, but recently, Rädecker et al*.*^6^ found increased parasitism (i.e., reduced transfer of photosynthates to the coral host) in algal symbionts prior to loss of algal symbiont cells from the coral host. Thus, it may be possible that the coral-algae symbiosis of *X. umbellata* in the present study was disrupted at the end of the experiment. A future experiment with similar experimental N and warming treatments and additional phosphate eutrophication could reveal if P starvation affects the resistance of *X. umbellata* to warming. For hard corals, moderate combined N and P eutrophication may even be beneficial under future ocean conditions^47^.

### Warming did not affect photophysiological parameters regardless of nitrate eutrophication

Algal symbiont cell density increased by 36%, although not significantly, under combined high nitrate eutrophication and warming compared to controls, whereas cell densities did not change in the warming treatment. Similarly, chl *a* content per algal symbiont cell and P_gross_ were not significantly affected by warming, regardless of nitrate eutrophication treatment. These results suggest that algal symbionts of *X. umbellata* were not negatively affected by the exposure to warming or combined warming and eutrophication.

Warming usually causes a loss of algal symbionts in *Xenia*^48,49^. *Xenia sp.* from the GBR showed highest loss of algal symbionts at 30 °C after only two days^48^ and *Xenia elongata* from the GBR was suggested as a biological indicator species for major bleaching events due to its high sensitivity to warming^49^. The superior thermal tolerance of *X. umbellata* from the northern Red Sea in the present study over *Xenia spp.* from the GBR concurs with predictions of previous studies ^35,50–52^, that corals of the northern Red Sea have especially high thermal tolerances, making this region a potential thermal refuge for coral reefs. Studies comparing the thermal tolerance of *X. umbellata* along the north–south gradient of the Red Sea (e.g., as Sawall et al*.*^53^) may reveal if the observed high thermal tolerance is caused by local adaptation or if it is a general trait of the species. Increasing algal symbiont cell densities are a common response to N eutrophication in corals, as N is often the limiting factor for algal symbiont growth^8^. Enhanced %N content and δ^15^N values of corals exposed to high nitrate eutrophication and warming at the end of the experiment suggest that nitrate was taken up, as dinitrogen fixation commonly leads to reduced δ^15^N^54^ and thus, assimilation of anthropogenic N can be traced through increasing δ^15^N^55^. Although %C values in the present study increased (not significantly) in the high eutrophication treatment after additional warming, possibly due to non-significant increases in algal symbiont cell densities, the C:N ratio was significantly reduced, further supporting *X. umbellata’s* incorporation of N from nitrate. Karcher et al*.*^56^ found reduced C:N values for xeniids, but not for turf algae or hard corals exposed to inorganic fertilizer. They concluded that soft corals may be more strongly affected by poor water quality due to their “luxury consumption”^57^ of N.

Coral tissue darkened with high nitrate eutrophication and warming, with no significant differences in algal symbiont cell density or chl *a* content. A similar observation was reported by Tilstra et al*.*^58^, who observed changes in colouration of *S. pistillata* colonies exposed to warming, without simultaneous changes in algal symbiont cell density or chl *a* content. Variation in colouration can also be caused by changing concentrations of the accessory pigment peridinin^59^, which can be affected by changing nutrient and temperature conditions in corals^45^. Additionally, non-significant changes in chl *a* contents, and algal symbiont cell densities, as well as protective algal pigments, especially conversions in xanthophyll pools, likely contributed to the change in colour^60^. Darkening of the tissue due to increases in algal symbiont cell densities after nitrate eutrophication has been observed for *S. pistillata*, which led to increased light absorbance^61^. Similarly, Fabricius^62^ found darker pigmentation of *Acropora millepora* in nutrient rich nearshore waters, and measured higher temperatures at their tissue surface, especially in areas with low water movement. Thus, darkening of corals in the present study could have caused increased water temperatures around the tissue surface through higher absorbance, potentially increasing thermal stress for *X. umbellata* with high nitrate eutrophication. The simultaneously reduced pulsation rate could have exacerbated this effect, as normal pulsation enhances mixing across the coral-water boundary layer^33^. Studies on pulsating soft corals should therefore monitor effects of pigmentation and pulsation rates on the corals' surface temperatures.

Nitrate concentrations of 15 μM in combination with warming reduced P_gross_ significantly for the hard coral *Porites fave*, when normalized to chl *a* content and algal symbiont cell density^63^. P_gross_ in the present study was standardized to surface area and non-significant increases in algal cell densities may have compensated for reduced per-cell photosynthesis, resulting in similar P_gross_ values to controls. This is especially likely considering the reduced P_gross_ observed before and shortly after the start of warming (day 16 and 22) with high nitrate eutrophication. Thus, P_gross_ was initially reduced by high nitrate eutrophication, but whole-colony photosynthesis was likely compensated by enhanced algal symbiont cell densities (though not significant) with additional warming. R remained stable between treatments throughout the study. In contrast, coral holobiont R increased with warming for *Orbicella faveolata*^22^ and *S. pistillata*, indicative for stress and increased energy demand^6^. Soft corals tend to have a higher heterotrophic capacity compared to hard corals, which likely alleviates their dependency on algal symbionts for metabolism during bleaching (Tremblay et al. 2016; Ferrier-Pagès et al. 2014; Grottoli et al. 2006; Fabricius & Klumpp 1995). However, R rates in the present study strongly correlated with P_gross_, suggesting that photosynthates were the main organic C source for R, despite the supply of zooplankton-containing coral food. The importance of heterotrophy in *Xenia* is not fully understood. Lewis^64^ found particulate matter in the coral's gastrovascular cavity and Vollstedt et al*.*^19^ found that *X. umbellata* fed with dissolved organic carbon (DOC) had higher thermal tolerance than starved colonies. Further investigation is needed to clarify if heterotrophic particle feeding similarly enhances thermal tolerance of *X. umbellata*.

Algal symbiont communities of the *X. umbellata* in the present study persisted despite potentially stressful conditions (as indicated by reduced pulsation rates, partial, and complete mortality) during high nitrate eutrophication and warming. Similar results were found for *X. elongata*, in which large numbers of algal symbiont cells were seen in necrotic tissue following exposure to a chemical dispersant^65^. Interestingly, some pulsating soft coral species have been reported to display within-colony algal symbiont migration into the gastrovascular cavity upon thermal stress, and thereby mitigating the bleaching response^66^. However, enhanced pigmentation measured in the tentacles of polyps in the present study is evidence against migration of algal symbionts into the gastrovascular cavity, as polyps of xeniids studied by Parrin et al*.*^67^ visibly paled due to migrating algal symbionts. Future studies are recommended to employ further microscopic analyses of host tissue to account for algal cell movement (e.g., as Parrin et al*.*^66^), as reuptake from the gastrovascular system could also provide insight into post-bleaching recovery and resilience^68^.

### Is *X. umbellata* more or less resistant to nitrate eutrophication and warming than hard corals?

Comparisons across eight similar experimental studies (all using nitrate as N source) on ten different hard coral species revealed that seven hard coral species were negatively affected by warming alone, whereas warming up to 32.8 °C over 22 days did not affect *X. umbellata* in the present study (Table 2). However, differences in temperature treatments, origins of the mother colonies, and other experimental conditions (e.g., feeding regime, P concentrations) between studies may have led to different outcomes. Nonetheless, *X. umbellata* appears to be less sensitive to warming than some reef building scleractinian corals, including *S. pistillata* from the northern Red Sea^23^. Overall, seven species were more negatively affected by combined nitrate eutrophication and warming than by warming alone in at least one response parameter. All of these previous studies used lower nitrate concentrations than the present study (< 37 μM) and seven of them were conducted over shorter experimental time periods. Six of the studies revealed reductions in photophysiology (algal symbiont cell density, chlorophyll content, photosynthesis), which were not significantly reduced by warming or the combined treatment in the present study. Therefore, while results of this study indicate that nitrate eutrophication can affect the otherwise high resistance of *X. umbellata* to warming, these impacts appear to be less than observed for a range of scleractinian corals.

**Table 2:** Experimental studies on effects of combined nitrate eutrophication and warming on hard corals in comparison to the present study. Only parameters similar to the ones measured in the present study were summarized. NO_3_ = Nitrate concentration, Warming = increase of temperature relative to controls, Fv/Fm = maximum quantum efficiency of Photosystem II. % values are changes relative to controls (* changes of imbalanced relative to nutrient replete conditions), n. s. = no significant difference to controls. Effect: " + " = combined warming and nitrate eutrophication result in greater reduction than warming alone; "−" = combined warming and nitrate eutrophication result in lesser reduction than warming alone; " = " = combined nitrate eutrophication and warming have similar effects to warming alone; "0" = no effects detected.

Study	Duration	Treatment	Coral species and origin	Parameter	Response	Effect
^63^	14 days	NO_3_: 15 μM Warming: 2 °C	*Porites cylindrica* Philippines	Algal symbiont cell density	n. s	0
				P_gross_	Warming: reduced by ~ 20% Warming + NO_3_: reduced by ~ 50%	+
				R	n. s	0
				Growth	n. s	0
^83^	30 days	NO_3_: 20 μM Warming: 5 °C	*Pocillopora damicornis* Gulf of Panama	Algal symbiont cell density	Warming: n. s Warming + NO_3_: reduced by ~ 50%	+
				Chl *a* content	Warming: n. s Warming + NO_3_: increase by ~ 100%	−
			*Porites lobata* Gulf of Panama	Algal symbiont cell density	Warming: reduced by ~ 30% Warming + NO_3_: reduced by ~ 30%	=
				Chl *a* content	n. s	0
^84^	One day	NO_3_: 5 μM Warming: 6 °C	*Turbinaria mesenteria* Vietnam	P_gross_	n. s	0
				R	n. s	0
				Mortality	Warming: 16.7% Warming + NO_3_: 33%	+
^20^	90 days	NO_3_: 4 μM Warming: 4 °C	*Acropora millepor,* Central Great Barrier Reef	Fv/Fm	n. s	0
				Skeletal growth	Warming: reduced by 45% Warming + NO_3_: reduced by 45%	=
				Mortality	n. s	0
			*Montipora tuberculosa* Central Great Barrier Reef	Fv/Fm	n. s	0
				Skeletal growth	n. s	0
				Mortality	n. s	0
^9^	10 days	NO_3_: ~ 2.7 μM Warming: 6 °C + light stress	*Acropora polystoma*	Algal symbiont cell density	Warming + light + NO_3_ + PO_4_: n. s Warming + light + NO_3_: reduced by 60%*	+
				Chl *a* content	n. s	0
				Fv/Fm	Warming + light + NO_3_ + PO_4_: dropped later below critical threshold Warming + light + NO_3_: dropped earlier	+
	20 days	NO_3_: ~ 2.7 μM Warming: 9 °C + light stress	*Acropora micro-phthalma*	Mortality	Warming + light + NO_3_ + PO_4_: 0% Warming + light + NO_3_: 100%*	+
^85^	Two days	NO_3_: 10 μM Warming: 5 °C	*Pocillopora damicornis* Japan	Algal symbiont cell density	Warming: reduced Warming + NO_3_: reduced, higher increase after recovery	−
				Chlorophyll content	Warming: n. s Warming + NO_3_: reduced after recovery	+
				Fv/Fm	Warming: reduced Warming + NO_3_: reduced, longer recovery period	+
^86^	Six days	NO_3_: 10 μM Warming: 5 °C	*Montipora digitata* Japan	Algal symbiont cell density	Warming: reduced by ~ 50% after 6 days Warming + NO_3_: reduced by ~ 50% after 3 days	+
				Fv/Fm	Warming: reduced by ~ 30% after 6 days Warming + NO_3_: reduced by ~ 30% after 6 days	=
^23^	35 days	NO_3_: 3 μM Warming: 5 °C	*Stylophora pistillata* Northern Red Sea	Algal symbiont cell density	Warming: reduced by 46% Warming + NO_3_: reduced by 33%	−
				Chlorophyll content	Warming: reduced by 36% Warming + NO_3_: reduced by 28%	−
				Fv/Fm	Warming: reduced by 31% Warming + NO_3_: reduced by 42%	+
				Growth (calcification)	Warming: reduced by 66% Warming + NO_3_: no effect	−
Present study (soft coral)	37 days, warming: 22 days	NO_3_: 6 μM / 37 μM Warming: 5 °C	*Xenia umbellata* Northern Red Sea	Algal symbiont cell density	n. s	0
				Chl *a* content	n. s	0
				P_gross_	n. s	0
				R	n. s	0
				Growth	Warming: n. s Warming + NO_3_: n. s. / partial mortality	0 / +
				Mortality	Warming: 0% Warming + NO_3_: 0% / 26%	0 / +

### What are the implications for coastal management?

In the present study, tank microcosms were enriched daily to nitrate concentrations of 6 µM and 37 µM, but measurements conducted only two to three hours later indicated nitrate concentrations of the water column averaging 2 µM and 23 µM, respectively. Thus, nitrate concentrations used in the present study represent daily nitrate input and not average nitrate concentrations during the whole experiment. In contrast, in situ nitrate measurements represent only what is present in the water column at a specific point of time, and are therefore not equivalent to nitrate input into the system due to rapid assimilation, for example by phytoplankton^69^. The finding of the present study that nitrate eutrophication as low as 2–6 μM can impact soft coral resistance to warming is relevant for the management of nearshore corals impacted by eutrophication. In the Red Sea for example, Ziegler et al*.*^70^ observed that soft corals, particularly xeniids, dominated reefs along the highly developed Jeddah coastline and Peña-García et al*.*^10^ measured total nitrogen (TN) concentrations of > 6 μM at these exact locations and concentrations of up to 2000 μM TN within the city bay. Nitrate composed on average 41% of TN in wastewater, making it the most common source of anthropogenic N at these sites. For the GBR, Gruber et al*.*^71^ reported highest nitrate + nitrite concentrations of 4.8 μM (300 μg L^−1^) near river mouths and up to 2.4 μM (150 μg L^−1^) at inshore reefs in the Tully region, with average nitrate + nitrite concentrations for the GBR below 1 μM. *Xenia* is one of the dominating soft coral genera on near shore reefs^72^ and upper mesophotic reefs^73^ of the GBR and was the only soft coral genus observed during a recent study in the Red Sea (El-Khaled et al., in press.). Additionally, *Xenia* was involved in hard coral to soft coral community shifts after blast fishing^31^ and an outbreak of the corallivore *Acanthaster planci*^74^. Moreover, Ziegler et al*.*^70^ reported highest abundance of *Xenia* at sites impacted by sedimentation and sewage discharge in the Red Sea (~ 12–15% vs. 0–3% at other sites). Although soft corals provide less structural complexity than hard corals^27,28^, they may still be a suitable habitat for many fish species^29^. Results of the present study indicate that soft coral populations may be severely impacted by the effects of combined nitrate eutrophication (of ≥ 2–6 μM) and warming. This can potentially lead to further degradation of these ecosystems towards dominance of macro- and turf algae, which often benefit from N eutrophication^56^. Thus, the results presented here support the conservation approach of enhancing coral resistance to global threats by managing local factors like inorganic N eutrophication^75,76^ for soft coral conservation. However, soft corals may be more resistant to nitrate eutrophication and warming than some hard coral taxa, which may facilitate community shifts from hard coral to soft coral dominance.

## Methods

### Experimental design and conditions

*Xenia umbellata* specimens used for the present study were collected from the northern Red Sea and maintained under aquarium conditions (salinity ~ 35 ‰, temperature ~ 27 °C) for over three years prior to the start of this experiment. Mother colonies from the maintenance aquarium were fragmented with sterile scalpels and attached to coral plugs (AF Plug Rocks, Aquaforest, Poland) with rubber bands. All mother colonies originated from the same genotype to reduce genotype-associated variation in the response to the experimental conditions. During a two-week acclimation period under ambient conditions, colonies were able to heal and grow onto coral plugs within the experimental tanks. Prior to the start of the experiment, a total of 168 colonies were randomly distributed among twelve experimental tanks (each 60 L). The tanks were separated into a technical part with heater, pump, and temperature logger (HOBO pendant temp/light, Onset, USA) and the experimental part. The experimental part was laid out with approximately 10 cm of sand five months before the start of the experiment to create a microcosm with microbial activity. Tanks were filled with 43 L of artificial seawater, prepared in a barrel with demineralized water and aquarium sea salt (Zoo Mix, Tropic Marin, Switzerland) to dissolve and reach required temperatures. In total, 14 coral colonies were placed on grid plateaus in each tank. Two light-emitting diode (LED) lamps (one Royal Blue matrix module and one Ultra Blue White matrix module, WALTRON daytime® LED light, Germany) were adjusted above each tank to guarantee equal light intensities measured in photosynthetically active radiation (PAR, Supplementary Table S1) with the LI-1400 Data Logger (LI-COR Biosciences, Germany) and day:night cycles of 12:12 h PAR was chosen to be close to conditions in the maintenance aquarium (~ 100 µmol photons m^-2^ s^−1^). The tanks were arranged in a three-level tower system with four tanks per level. The four treatments were distributed in an approximate latin square design, with each treatment in every level (Fig. 1). The coral colonies were fed with dried marine plankton (Reef-Roids, Polyp Lab, USA) at concentrations of 10 mg L^−1^ twice a week throughout the experiment to keep conditions close to the previous maintenance aquarium. Pumps were turned off for 30 min during feeding. The twelve tanks were connected to form one system with continuous water through-flow, and separated on day one of the experiment to ensure equal water quality among treatments at the start of the experiment. During the experiment, oxygen, pH, salinity, and temperature were measured daily and salinity and temperatures were adjusted when necessary. Chemical water parameters for all tanks were maintained at equal conditions (Supplementary Table S1) through regular water exchange of 10–20%. Tanks were cleaned every one to two weeks to remove any biofouling.

### Experimental nitrate and temperature treatments

Nitrate was adjusted to medium (6 μM) and high (37 μM) concentrations, which are comparable to previous nitrate eutrophication experiments with corals^9,13,20^ and in situ conditions around coastal metropolitan areas in the Red Sea^10^. Each treatment was replicated in three tanks while six other tanks were kept at low nitrate concentrations (~ 0.6 μM). These were divided into three controls (LN) and three tanks with additional warming from day 17 (LN + W, Fig. 1). Nitrate solutions were prepared from sodium nitrate (NaNO_3_) and demineralized water before every addition. Nitrate concentrations were measured twice a week photometrically (Supplementary Figure S2). Briefly, 100 mg zinc dust and 1 mL cadmium-sulphate solution (CdSO_4_ × 8 H_2_O) were added to 10 mL water samples to reduce nitrate to nitrite. Subsequently, 0.05 mL sulphanilamide-solution (C_6_H_8_N_2_O_2_S) and 0.05 mL N-(1-naphthyl) ethylenediamine dihydrochloride) were added. The resulting change in colouration is linear to the nitrate concentration and was measured with a photometer after calibration (Trilogy, Turner Designs, USA). Nitrate was added once on day one and daily from day five of the experiment, as nitrate was taken up rapidly from the water column. For medium concentrations (6 μM), it was assumed that nitrate was reduced to ambient concentrations within a day, as concentrations in high treatment tanks were reduced by 24.6 ± 9.1 μM per day. From day eleven, nitrate concentrations in the high eutrophication treatments were measured daily and adjusted to the aimed concentration. Temperatures fluctuated by ~ 1 °C on a daily basis due to additional heat created by the LED lamps. Temperatures were equal among all tanks for the first 16 days, averaging 27.7 ± 0.7 °C and fluctuating between 26.1 °C and 29.3 °C due to weather conditions affecting the indoor temperatures (Fig. 1). From day 17, temperatures in all but three control tanks (LN) were gradually increased and reached 32.8 ± 0.3 °C on day 37. Control tanks (LN) were not experimentally warmed and stayed within the initial temperature range until day 35, when the temperature in one control tank increased to 30 °C, and on day 37 to 30.7 °C due to the heat coming from adjacent tanks.

### Growth rate and survival measurement

The polyps of three marked coral colonies were counted by the same observer every five to eleven days and percent growth rates were calculated as the change in total number of polyps which were counted (*p*) standardized to the number of days (*d*) that have passed since the last measurement relative to the initial number of counted polyps (Formula 1).

1 $$Growth\ rate= \left(\frac{\left(\frac{\left({p}_{end}- {p}_{start}\right)}{d}\right)}{{p}_{start}}\right)*100$$

Negative growth rates were defined as partial mortality (i.e., mortality of some colony polyps), which is a characteristic of modular colonial organisms like corals, where parts of their living tissue can die off without causing whole-colony mortality^77^. Mean growth rates of three colonies were calculated per tank. *Xenia umbellata* colonies were individually placed into glass jars within the experimental tanks without exposing them to air, then the jars were removed from the tanks and kept in tempered water baths of the same temperature as the respective experimental tank to avoid stress from sudden temperature change prior to counting. Soft tweezers were used to spread and count the polyps. Colony survival of all *X. umbellata* colonies present in the experimental tanks was determined daily by checking for polyp movement. Corals that did not show movement were touched with soft tweezers to test for a reaction. Unresponsive colonies were defined as dead and removed from the tanks.

### Pulsation rate measurement

Pulsation rate as a proxy for coral health was counted following the method developed by Vollstedt et al*.*^19^. Briefly, pulsation rates of three randomly selected polyps from three marked colonies per experimental tank were measured by the same observer at noon, before addition of coral food, and every six to eight days, starting on day one of the experiment. Mean rates were calculated for each colony and subsequently, for each tank, resulting in three replicate values per treatment. Pulsations were counted within a time frame of 30 s and standardized to one minute with one pulsation defined as one whole contraction of the polyp (open—fully closed—open). Incomplete contractions were not counted. Upon mortality of marked colonies, new colonies from the same tank were allocated to pulsation measurements.

### Gross photosynthesis and respiration measurements

Every six to eight days, starting on day one of the experiment, one marked colony per tank (same colony measured each time point, n = 3) was placed into 160-mL gas tight jars submerged in the experimental tanks to avoid stress from air exposure. Jars were removed from the experimental tanks, closed without capturing air and incubated for 90 to 120 min in the light (135 ± 4 µmol photons m^-2^ s^−1^ PAR) and in darkness in tempered water baths of the same temperature as the respective experimental tank. Stirring bars in the jars ensured homogenous oxygen concentrations. Oxygen concentrations were measured with an optode sensor (HACH LDO, HACH HQ 40d, Hach Lange, Germany) before and after each incubation and the start concentration was subtracted from the end concentration to eventually calculate oxygen fluxes (*oxy*), which was defined as net photosynthesis (P_net_) in the light, and R in the dark. Values were normalized to incubation time (*h*). Biofouling on the plug was carefully removed with a soft brush prior to being placed in the jars, though to account for any remaining biofilm, one blank plug was placed in every tank during the experiment and used for blank P_net_ and R measurements. For every measurement day, one to two tanks were randomly chosen for blank incubations and additional blank incubations were conducted on day 35. The average of all blank oxygen fluxes (n = 24, *blank*) standardized to time of incubation (*h*) was subtracted from the coral incubations for each, light and dark incubations, as there was no significant difference in blank fluxes between treatments (1-way ANOVA; Light: F = 0.445, *p* = 0.775; Dark: F = 2.029, *P* = 0.131). After the incubations, the number of polyps per colony (*p*) was multiplied by the average surface area (*s*) of one *X. umbellata* polyp^44^ to normalize oxygen fluxes to colony surface area. This method was established by Bednarz et al*.*^44^ for *Xenia*. Measurements of the polyps were taken from pictures using the software ImageJ (1.53e, Wayne Rasband and contributors, National Institutes of Health, USA) to reduce stress on the corals and to avoid polyp retraction affecting the measurement. In this way, 80 random polyps from 18 colonies used in the experiment were measured. Finally, all values were normalized to the volume of the incubation jars (*v,* Formula 2).

2 $$P_{{net(light)}} \;{\text{or}}R_{{(dark)}} = \frac{{\left( {\frac{{\left( {oxy_{{(light\;{\text{or}}\;dark{\text{ }}}} } \right)}}{h} - \frac{{\left( {blank_{{(light\;{\text{or}}\;dark}} } \right)}}{h}} \right)}}{{p*s*v}}$$

Gross photosynthesis (P_gross_) was calculated with formula (3).

3 $${P}_{gross}={ P}_{net}+ \left|R\right|$$

### Sample processing for algal symbiont cell density and chlorophyll a measurement

Methods for soft coral sample processing and normalisation metrics were adopted as recommended by Pupier et al*.*^42^. Briefly, on day 1, 15, and 37, one colony per tank (i.e. three colonies per treatment) was removed from its coral plug, any biofouling was removed, and finally stored in plastic bags and frozen at −20 °C. All samples were then freeze-dried at −60 °C for 24 h and stored in the dark pending analysis. Dry weight (DW) of each sample was used as the normalisation metric for algal symbiont cell density. Samples were homogenised in distilled water, as Pupier et al*.*^42^ found no differences in algal symbiont cell densities when samples were prepared with distilled water or filtered seawater. The tissue slurry was used for algal cell density counts and chl *a* measurement.

To separate coral tissue and algal cells in the tissue slurry, subsamples were centrifuged for 10 min, supernatant discarded, pellet re-suspended in 2 mL distilled water, centrifuged again for 10 min and the supernatant discarded. The pellet was re-suspended in 2 mL distilled water, thoroughly mixed and transferred onto two grids of one haemocytometer (Improved Neubauer counting chamber, depth 0.1 mm) allowing for two replicate counts per sample. For algal symbiont counts, the standardized haemocytometer counting method described by LeGresley & McDermott^78^ was used.

Subsamples for the chl *a* concentration measurement were rinsed twice by centrifugation as described previously. The remaining pellet was re-suspended in 2 mL 100% acetone for chlorophyll extraction, and kept in darkness for 24 h at 4 °C. Under minimal light exposure, the extraction sample was centrifuged for five minutes and then transferred into two quartz cuvettes, allowing for two replicate readings per sample. Chl *a* concentration measurements were conducted using a UV-Spectrophotometer (GENESYS 150, Fisher Scientific, Germany), following the method for dinoflagellates described by Jeffrey & Humphrey^79^. Resulting concentrations were standardized to host DW and subsequently, to algal symbiont cell density to calculate cellular chl *a* content.

### Quantification of coral colouration

Photographs of three colonies per tank were taken over the course of the experiment. Documenting the same colonies was important for establishing changes over time. Photographs were taken under white light with an Olympus TG6 underwater camera with fixed manual settings (ISO 100, f/1.4, × 4 magnification). A colour standard was used for later white balance adjustment in Adobe Photoshop CS6. One marked colony per high nitrate enriched tank was used to create colour reference cards similar to the method by Siebeck et al*.*^59^, who identified brightness, saturation and hues that correlated with algal symbiont densities and chl *a* content of hard corals to monitor coral bleaching. Since *X. umbellata* lacks a calcium carbonate skeleton that can act as a white contrast when algal symbionts are removed from host tissue, red, green, and blue (RGB) pixel values were used in the present study to assess overall colouration change. Briefly, for each coral, five polyps were randomly selected and RGB values (25 × 25 pixel square) obtained from their tentacles. Previous studies have found algal densities frequently higher in tentacles and tentacle tips^80^, thus these areas are likely prone to colouration change. The resulting range of RGB values (Supplementary Figure S3) was used to identify five colour scores by #HEX colour codes (Supplementary Table S2) which represented the change in colouration from one (initial colour) to five (most darkened). Using these colour references, the colour score of three marked colonies per tank was identified by one observer from pictures taken as described above. Mean colour scores were calculated per tank, resulting in three replicates per treatment.

### Carbon and nitrogen elemental, and stable isotope analysis

Colonies for elemental analysis were randomly chosen from each tank and removed from coral plugs, stored in plastic bags and frozen at − 20 °C pending analysis. *X. umbellata* colonies were dried in an oven for 48 h at 40 °C until weight consistency was reached, then grinded with a mortar and pestle, and the tissue powder was transferred into tin cups. C and N quantities and stable isotope ratios were analysed as described in Karcher et al*.*^56^. Isotopic ratios (*r*) are shown as the ratio of heavier:lighter isotope (^13^C:^12^C or ^15^ N:^14^ N) and notated as either δ^13^C or δ^15^N (‰) using formula 4:

4 $$\delta X =\left(\frac{{r}_{sample}}{{r}_{reference}}-1\right)*1000$$

where *r*_reference_ is Vienna Pee Dee Belemnite for δ^13^C (0.01118) and atmospheric N for δ^15^N (0.00368).

### Statistical analyses

All data was presented as means with error bars representing standard deviations, and the alpha levels for all statistical tests were set to *p* = 0.05. To test for significant effects of treatments over time for randomly collected data (Cell density, chl *a* & elemental stoichiometry), 2-way analyses of variance (ANOVA) were conducted, and non-normally distributed data (δ^15^N) were rank-transformed and analysed using non-parametric approaches (ARTool package), as proposed by Feys^81^. For data obtained from the repeated measurements P_gross_, R, and growth rate, a 2-way mixed-model ANOVA was conducted with 'day' as within-subject factor and 'treatment' as between-subject factor. Normality was tested with the Shapiro–Wilk test, homogeneity of variance was tested with the Levene's test, and no outliers were identified (rstatix package). Box's M-test was used to confirm homogeneity of covariance, and sphericity was tested with the Mauchly's test and corrected with the Greenhouse–Geisser sphericity correction when violated. For non-parametric data of repeated measurements (survival, pulsation rates, colour scores), non-parametric mixed-effects models were conducted using the R package 'nparLD'^82^. For *post-hoc* analysis, pairwise comparisons (pwc) with Bonferroni adjustment were used, with *t*-test for parametric and Dunn's test for non-parametric data. No *post-hoc* test could be conducted for survival data, as there was no variance within most groups. Day one was excluded to enable *post-hoc* tests for colour score data, because all groups had identical values. Spearman’s correlation was run to test for the relationship between P_gross_ and R.

## Supplementary Information

Supplementary Information 1.

Supplementary Information 2.

## Data Availability

Raw data of the current study is included in this published article (Supplementary Table S3).
