# Supplementary material for: The pulsating soft coral Xenia umbellata shows high resistance to warming when nitrate concentrations are low
Source: Sci Rep. 2022 Oct 6;12:16788. doi: 10.1038/s41598-022-21110-w (PMC9537297; doi:10.1038/s41598-022-21110-w)
Supplement: Supplementary file 1 — Supplementary Information 1. [file 41598_2022_21110_MOESM1_ESM.docx]

**The pulsating soft coral** ***Xenia umbellata* shows high resistance to warming when nitrate concentrations are low**

Bianca Thobor^1*^, Arjen Tilstra^1^, David G. Bourne^2,3^, Karin Springer^4^, Selma Deborah Mezger^1^, Ulrich Struck^5,6^, Franziska Bockelmann^1^, Lisa Zimmermann^1^, Ana Belén Yánez Suárez^1^, Annabell Klinke^1^, and Christian Wild^1^

^1^University of Bremen, Faculty of Biology and Chemistry, Department of Marine Ecology, UFT building, Leobener Str. 6, 28359 Bremen, Germany

^2^James Cook University, College of Science and Engineering, 1 Angus Smith Drive, Douglas, QLD, 4814, Australia

^3^Australian Institute of Marine Science, Cape Ferguson, Townsville, QLD, 4810

^4^University of Bremen, Faculty of Biology and Chemistry, Marine Botany, NW2 building, Leobener Str. 5, 28359 Bremen, Germany

^5^Museum für Naturkunde, Leibniz Institute for Evolution and Biodiversity Science, Invalidenstr. 43, 10115 Berlin, Germany

^6^Free University Berlin, Department of Earth Sciences, Malteserstr 74-100, Haus D, 12249 Berlin, Germany

*thobor@uni-bremen.de

**Supplementary data**

Table S1 Water quality parameters of experimental tanks per treatment during the experiment. Mean values ± standard deviations for all tanks and measurements. Nitrate concentrations were measured two to three hours after nitrate enrichment to 6 µM (medium) and 37 µM (high).

| **Variable** | **LN** | **LN + W** | **MN + W** | **HN + W** | **n** |
| --- | --- | --- | --- | --- | --- |
| **NO_3_** (μM) | 0.30 ± 0.42 | 0.51 ± 0.67 | 2.33 ± 1.47 | 23.40 ± 12.54 | 33 |
| **PAR**(µmol m^-^² s^-1^) | 103.5 ± 7.0 | 112.8 ± 9.6 | 108.3 ± 10.3 | 98.2 ± 10.1 | 9 |
| **O_2_** (mg L^-1^) | 6.3 ± 0.8 | 6.2 ± 0.8 | 6.3 ± 1.1 | 6.6 ± 1.3 | 111 |
| **Salinity** (‰) | 35.4 ± 0.7 | 35.2 ± 0.5 | 34.8 ± 0.5 | 35.1 ± 0.4 | 111 |
| pH | 8.52 ± 0.49 | 8.54 ± 0.36 | 8.53 ± 0.28 | 8.60 ± 0.54 | 111 |
| **NO_2_** (mg L^-1^) | <0.01 | <0.01 | <0.01 | 0.03 ± 0.09 | 111 |
| **KH** | 6.9 ± 1.1 | 6.7 ± 1.0 | 6.8 ± 1.0 | 6.6 ± 1.3 | 30 |
| **NH_4_** (mg L^-1^) | *Always below detection limi*t (<0.05) | | | | 30 |
| **PO_4_** (μM) | *Always below detection limit* (<0.21) | | | | 30 |
| **SiO_4_** (mg L^-1^) | 0.36 ± 0.26 | 0.43 ± 0.27 | 0.27 ± 0.18 | 0.37 ± 0.39 | 30 |
| **Mg** (mg L^-1^) | 1316 ± 204 | 1312 ± 162 | 1316 ± 170 | 1352 ± 193 | 30 |
| **Ca** (mg L^-1^) | 401 ± 24 | 401 ± 23 | 387 ± 21 | 388 ± 21 | 30 |

Table S2 Definitions of colour scores by #HEX codes and red, green, and blue (RGB) values.

| **Score** | **1** | **2** | **3** | **4** | **5** |
| --- | --- | --- | --- | --- | --- |
| #HEX code | #b69482 | #b39e92 | #b0a8a2 | #bdb2b2 | #abbdc3 |
| **Red** | 182 | 179 | 176 | 173 | 171 |
| **Green** | 148 | 158 | 168 | 178 | 189 |
| **Blue** | 130 | 146 | 162 | 178 | 195 |

Table S3 Supplementary raw data of the present study, sorted by corresponding figure number. *Xenia umbellata* colonies were held for 37 days in control tanks with low nitrate (LN, ~0.6 μM) and three treatments: LN + W = low nitrate (~0.6 μM) + warming from day 17; MN + W = medium nitrate eutrophication (~6 μM) + warming from day 17; HN + W = high nitrate eutrophication (~37 μM) + warming from day 17. Measurements were taken from colonies of three replicate tanks per treatment. Where several measurements were taken from the same tank (growth rate, pulsation rate, colour scores), these are reported before and after the average of each tank was calculated.


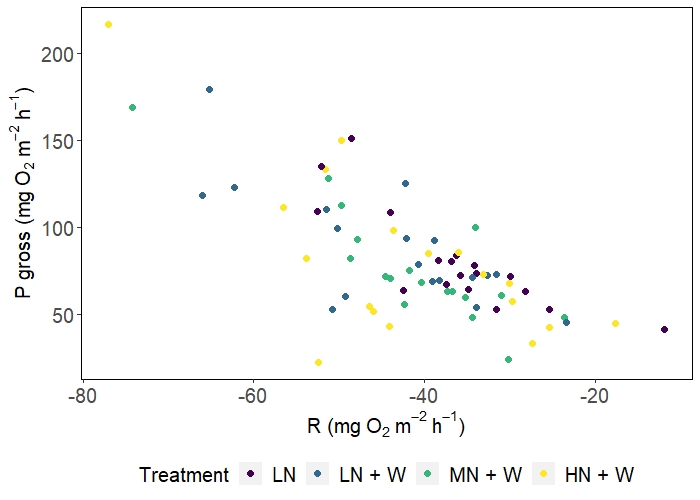


Figure S1 Correlation of gross photosynthesis (P_gross_) and respiration (R) of *Xenia umbellata* colonies from control tanks with low nitrate (LN, ~0.6 μM) and three treatments: LN + W = low nitrate (~0.6 μM) + warming from day 17; MN + W = medium nitrate eutrophication (~6 μM) + warming from day 17; HN + W = high nitrate eutrophication (~37 μM) + warming from day 17. Summary of all measurements conducted during the experiment. Significant negative Spearman’s correlation: *r_S_* = -0.63, n = 72, p < 0.001.


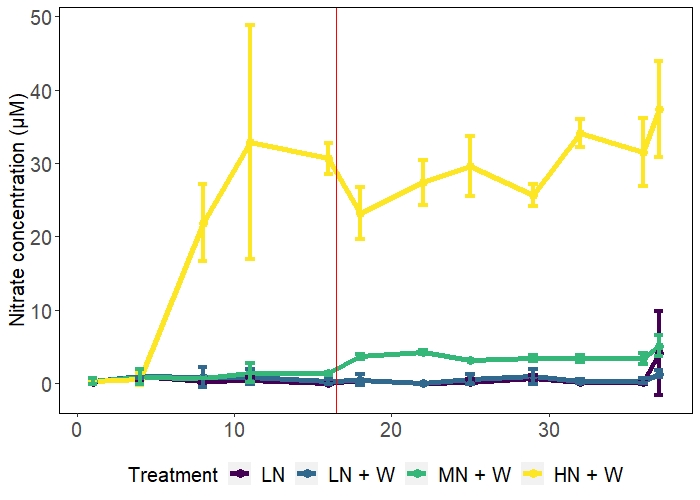


Figure S2 Nitrate concentrations (µM) measured in tanks before the addition of new nitrate by adjusting concentrations to 6 µM in medium nitrate (MN + W), and to 37 µM in high nitrate (HN + W) treatments. No nitrate was added to the low nitrate treatment (LN), and the low nitrate treatment with additional warming (LN + W).


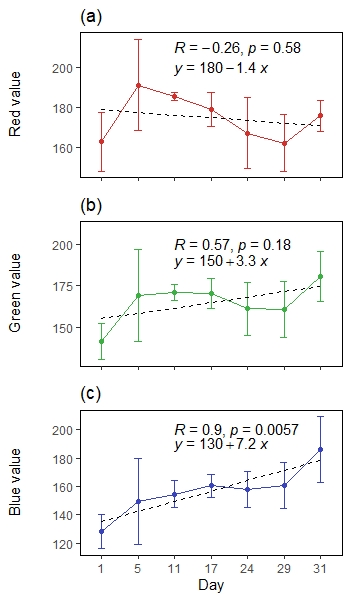


Figure S3 Changes in red (a), green (b), and blue (c) values derived from photograph analysis of *Xenia umbellata* colonies exposed to high nitrate eutrophication (37 µM) and gradual warming (HN + W) throughout the experiment. Error bars represent standard deviations of three replicates. Dashed lines represent linear regression line, where R > 0.5 indicates correlation between colour value and day of the experiment. Values were used to identify five colour scores (Table 2).
